# Avian antimicrobial peptides: in vitro and in ovo characterization and protection from early chick mortality caused by yolk sac infection

**DOI:** 10.1038/s41598-021-81734-2

**Published:** 2021-01-22

**Authors:** Thuy Thi Thu Nguyen, Brenda Allan, Colette Wheler, Wolfgang Köster, Volker Gerdts, Arshud Dar

**Affiliations:** Vaccine and Infectious Disease Organization (VIDO), University of Saskatchewan, 120-Veterinary Road, Saskatoon, SK S7N 5E3 Canada

**Keywords:** Drug discovery, Immunology, Microbiology, Diseases, Risk factors

## Abstract

Increasing antibiotic resistance is a matter of grave concern for consumers, public health authorities, farmers, and researchers. Antimicrobial peptides (AMPs) are emerging as novel and effective non-antibiotic tools to combat infectious diseases in poultry. In this study, we evaluated six avian AMPs including 2 truncated cathelicidins, [CATH-1(6–26) and CATH-2(1–15)], and 4 avian β-defensins (ABD1, 2, 6 and 9) for their bactericidal and immunomodulatory activities. Our findings have shown CATH-1(6–26) and ABD1 being the two most potent avian AMPs effective against Gram-positive and Gram-negative bacteria investigated in these studies. Moreover, CATH-1(6–26) inhibited LPS-induced NO production and exhibited dose-dependent cytotoxicity to HD11 cells. While, ABD1 blocked LPS-induced IL-1β gene induction and was non-toxic to HD11 cells. Importantly, in ovo administration of these AMPs demonstrated that ABD1 can offer significant protection from early chick mortality (44% less mortality in ABD1 treated group versus the control group) due to the experimental yolk sac infection caused by avian pathogenic *Escherichia coli*. Our data suggest that in ovo administration of ABD1 has immunomodulatory and anti-infection activity comparable with CpG ODN. Thus, ABD1 can be a significant addition to potential alternatives to antibiotics for the control of bacterial infections in young chicks.

## Introduction

Excessive and non-judicious use of antibiotics in medicine and agriculture has been associated with rapid emergence and distribution of multidrug-resistant pathogens in human and animal populations^1^. Importantly, numbers of pathogenic and/or non-pathogenic organisms in chickens including *Escherichia coli*, *Salmonella *sp., *Campylobacter *sp., and *Clostridium perfringens* are linked with economically important poultry diseases and food-borne illnesses in humans. While, these organisms have shown multiple drug resistance (MDR) leading to reduced antibiotics efficacy, increased economic losses, and critical side effects due to long-term treatments and multiple drug usage^2–5^. Therefore, identification and characterization of alternatives to antibiotics possessing anti-infective, immune-modulating, and growth-promoting abilities are expected to replace the use of antibiotics in poultry production leading to the provision of antibiotic-free safe food to consumers.

Since the first discovery of gramicidin from bacteria in 1939 and defensin from animals in 1956 (isolated from rabbit leukocytes), the therapeutic application of antimicrobial peptides (AMPs) has emerged as an attractive research area in medicine and animal production. According to the dbAMP databases, to date, more than 4271 AMPs have been experimentally validated, whereas, more than 8118 predicted AMPs are still pending for biological characterization (http://140.138.77.240/~dbamp/index.php). Antimicrobial peptides have a broad spectrum of antimicrobial, immunomodulatory, wound healing, anti-biofilm, and anti-cancer cell activities with emergence of significantly lower resistance rates^6,7^. AMPs are small peptides (20–50 amino acids) that serve as conserved components of the innate immunity in living species including bacteria, insects, plants, and animals^6^. AMPs can be classified into α-helical, β-sheet, or peptides with extended/random-coil based secondary structures^8^. Cationic AMPs have a positive net charge (+ 2 to + 11) with ~ 50% hydrophobic residues. Mechanistically, positive charge and hydrophobicity allow peptides to bind and penetrate into the bacterial membrane with formation of transmembrane pores and ion channels resulting inhibition of intracellular functions (DNA replication, protein synthesis, and protein function) causing cell lysis and death^9,10^. Moreover, AMPs may eliminate pathogens via recruitment and activation of leukocytes, enhancement of auto-inflammation, phagocytosis, and neutralization of toxic bacterial products (lipopolysaccharide-LPS, lipoteichoic acid-LTA)^11,12^.

Recently, many avian AMPs have been investigated for their antimicrobial and immune-modulatory activities. Cathelicidins (CATH) and β-defensins represent two major families of avian AMPs which are derived from the bone marrow and/or epithelial cells and expressed in various tissues^7,13,14^. Avian cathelicidins are classified into four classes namely CATH-1, CATH-2, CATH-3^15^, and CATH-B1^16^. CATH-1 and CATH-2 have shown broad-spectrum antimicrobial activities against Gram-positive and -negative bacteria^15^. While, fourteen distinct β-defensin, genes (namely AvBD 1–14) with a highly conserved N-terminal signal peptide have been identified in chickens^7^. AvBD1 and AvBD2 derived from chicken leukocytes have exhibited antimicrobial activities against *E. coli*, *Listeria monocytogenes,* and *Candida albicans*^17^. Similarly, recombinant AvBD6 has moderate antimicrobial activities against *Salmonella* Typhimurium and *Salmonella* Enteritidis^18^, whereas, synthetic and recombinant AvBD9 have been found effective against some bacteria with minimal inhibitory concentration (MIC) ranging from 8 to 64 μM^19^.

The prophylactic or therapeutic application of AMPs is limited due to their higher production cost, protease susceptibility, and potential toxicity^7^. Therefore, various approaches including the characterization of short peptides, introduction of peptide amidation or cyclization, and the use of liposome encapsulation as a delivery system have been investigated to improve peptide stability and efficacy and to reduced cost and toxicity^6,20^. A truncated peptide of CATH-1, named CATH-1(6–26), has exhibited highly effective antimicrobial activities against a wide variety of bacteria^15^ along with lipopolysaccharide (LPS)-neutralization ability and substantially reduced cytotoxicity^21^. Moreover, CATH-1(6–26) has offered over 50% protection from *Staphylococcus aureus* (MRSA) lethal infection in mice^22^. Immunologically, this truncated peptide enhanced neutrophil recruitment and activated macrophages by inducing the expression of inflammatory mediators including IL-1β, CCL2, and CCL3^23^. While, the truncated CATH-2(1–15) has exhibited a potent antimicrobial activity associated with low cytotoxicity towards chicken erythrocytes and human peripheral blood mononuclear cells (PBMCs)^24^.

This study was designed to evaluate and compare in vitro antibacterial activities of two truncated cathelicidins including CATH-1(6–26) and CATH-2(1–15), and four avian β-defensins (ABD1, ABD2, ABD6, ABD9) against bacterial poultry pathogens important for economic, public health and food safety aspects of poultry production. Moreover, the cytotoxicity, immune-related gene expression, and nitric oxide (NO) production was also investigated to understand the immunomodulatory effects of these peptides. Importantly, protective activities of selected avian AMPs were investigated following in ovo administration of these AMPs. The protective potential of the AMPs was evaluated through assessing reduction in early chick mortalities (ECM) due to experimental yolk-sac infection (YSI) caused by an avian pathogenic *E. coli* (APEC) infection in young chicks. Moreover, the transcriptional regulation of some immune-related genes in the spleens of treated chicks was investigated to understand the molecular basis of protection offered by the peptides used in these studies. Our data suggest that some of these peptides may serve as potential alternatives to antibiotics.

## Results

### Avian antimicrobial peptides

Working stocks for in vitro and in ovo experiments were prepared by dissolving lyophilised powder of the linear forms of peptides (with > 95% purity assessed by MS) in LPS-free water (molecular water, Sigma). Physiochemical properties of peptides used in these studies are shown in Table 1. Two truncated cathelicidins, CATH-1(6–26) and CATH-2(1–15) have short lengths with 21 amino acids (aa) (2.5 kDa) and 15 aa (Mw = 2.03 kDa), respectively, while, four avian beta-defensins (ABD1, 2, 6 and 9) were synthesized as full-length mature peptides ranging from 39 to 42 aa (4.2–4.7 kDa). The net charge varied from 4 to 8 with a net charge of 8 for CATH-2(1–15) and ABD1, while ABD2 and ABD9 have a net charge of 4. CATH-1(6–26), CATH-2(1–15), and ABD1 have higher pIs compared with three other ABDs. In addition, hydrophobicity was noted (in decreasing order) as ABD2 (0.616), ABD1 (0.547), CATH-1(6–26) (0.544), and ABD6 (0.504); whereas CATH-2(1–15) and ABD9 have the lowest hydrophobicity at 0.103 and 0.396, respectively. The hydrophobic moment as a quantitative measure of the amphiphilicity of AMPs exhibited the highest value for CATH-2(1–15) (0.585 µM); medium values for CATH-1(6–26) (0.28 µM), ABD1 (0.181 µM), and ABD6 (0.117 µM); whereas the lowest values were found for ABD2 (0.047 µM) and ABD9 (0.094 µM).

**Table 1:** Physiochemical properties and predicted secondary structures of six avian peptides used in this study.

Peptide name	Amino acid sequence	Length	Net charge	Mw (Da)	pI	Hydrophobicity (H)	Hydrophobic moment (µM)
CATH-1(6–26)	WPLVIRTVIAGYNLYRAIKKK-NH2	21	5	2503	11.02	0.544	0.28
CATH-2(1–15)	RFGRFLRKIRRFRPK	15	8	2033	12.81	0.103	0.585
ABD1	GRKSDCFRKSGFCAFLKCPSLTLISGKCSRFYLCCKRIW	39	8	4510	10	0.547	0.181
ABD2	RDMLFCKGGSCHFGGCPSHLIKVGSCFGFRSCCKWPWNA	39	4	4324	8.38	0.616	0.047
ABD6	SPIHACRYQRGVCIPGPCRWPYYRVGSCGSGLKSCCVRNRWA	42	7	4744	9.43	0.504	0.117
ABD9	DTLACRQSHGSCSFVACRAPSVDIGTCRGGKLKCCKWAPSS	41	4	4288	8.42	0.396	0.094

α-helical structures, random-coil, and extended-strand are indicated as letters h, c, and e, respectively.

**Figure 1:**
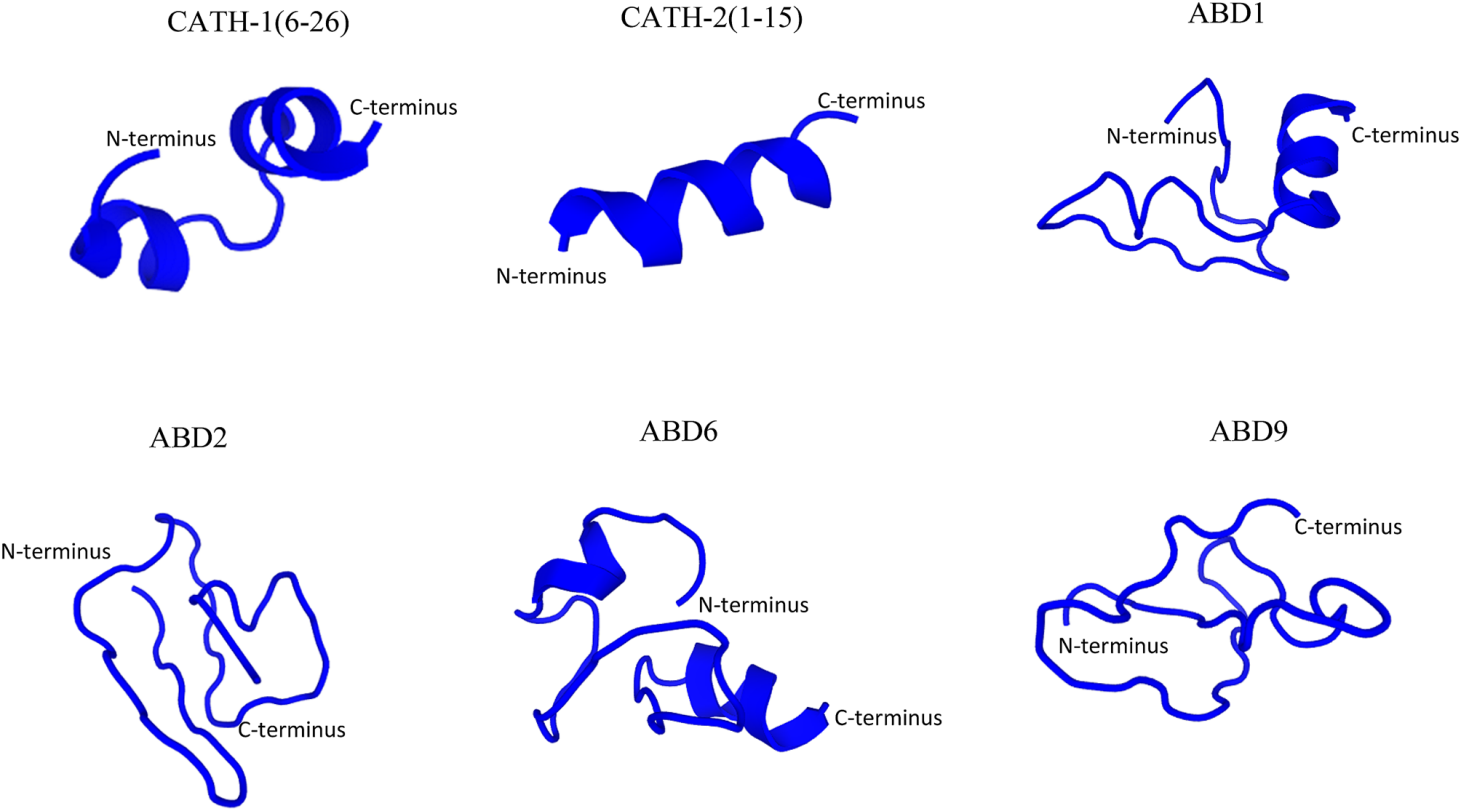
Three-dimensional structures of the antimicrobial peptides used in this study. PEP-FOLD3 program from “RPBS Web Portal” was used to generate the snapshots of linear peptides in an aqueous solution. The structures were represented as cartoons.

The predicted tertiary structures of peptides are shown in Fig. 1. CATH-1(6–26), CATH-2(1–15), ABD1, and ABD6 represent α-helical peptides, while ABD2 and ABD9 reflect extended/random-coil structures. CATH-1(6–26) and ABD6 are characterized by two short α-helical fragments, while CATH-2(1–15) appears as an α-helix of almost the whole peptide. ABD1 forms an α-helix on the C-terminal side while the N-terminal seems more loosely organised.

### In vitro antibacterial activity

**Figure 2:**
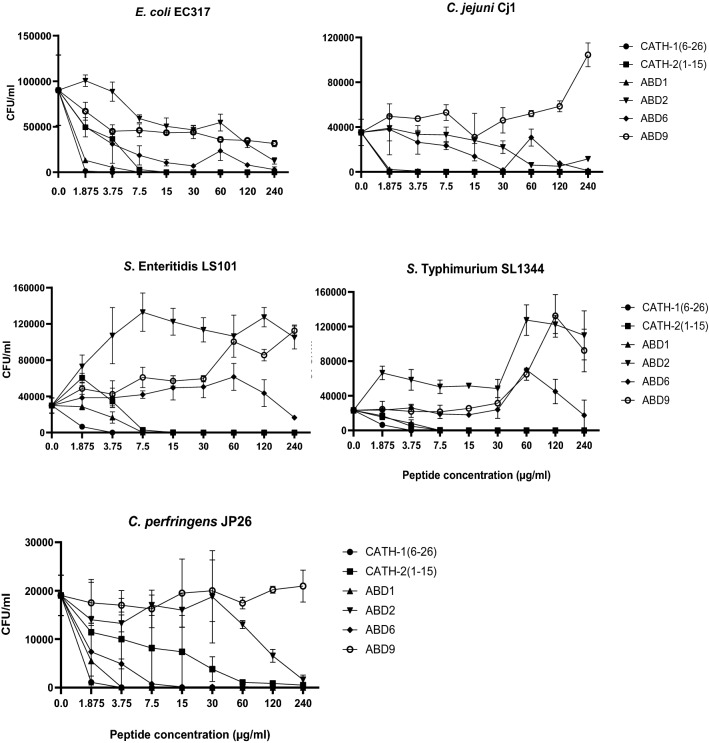
Antibacterial activities of 6 synthesized peptides against five important pathogenic bacteria isolated from birds. In each experiment, respective bacterial species (from 10^4^ to 10^5^ CFU/ml) were incubated for 2 h with peptide concentrations ranging from 1.875 to 240 µg/ml in duplicate, serially diluted, and plated on agar media. The data is shown as mean ± SD of at least two independent experiments on different days.

Six synthesized peptides have shown various degrees of antibacterial activities against five bacterial strains including *E. coli*, *S.* Enteritidis, *S*. Typhimurium, *C. jejuni*, and *C. perfringens* originated from poultry (Fig. 2; Table 2). Amongst all peptides, CATH-1(6–26) and ABD1 demonstrated the highest bactericidal effects, followed by CATH-2(1–15). While ABD2, 6, and 9 displayed lower inhibitory activities against the tested bacterial strains. Truncated CATH-1(6–26) was able to inhibit the bacterial growth of all tested bacteria with the minimal inhibitory concentration (MIC) = 1.5 µM or less. Similarly, ABD1 killed all pathogens except *C. perfringens* with MIC = 1.66 µM, the MIC for *C. perfringens* was 0.83 µM. The MIC of CATH-2(1–15) varied depending upon targeted bacterial strains. For instance, CATH-2(1–15) inhibited *C. jejuni* strain *Cj1* at MIC = 0.92 µM, while, MIC = 7.38 µM was observed for other Gram-negative strains. Moreover, this peptide [CATH-2(1–15)] displayed a lower bactericidal activity for *C. perfringens* JP26, with MIC > 118.05 µM. ABD6 was able to kill *C. perfringens* JP26 with MIC = 50.59 µM, whereas, it has shown no or very low inhibition against Gram-negative bacterial strains tested here. ABD2 had moderate effects on *E. coli*, *C. jejuni*, and *C. perfringens* at high doses (60–240 µg/ml), while the peptide displayed no effect on *S.* Enteritidis and *S.* Typhimurium. ABD9 has shown ~ 40–90% growth inhibition of *E. coli* strain EC317 within the dosage range of 30 – 240 µg/ml, whereas, negative effects were noted in killing assays against bacterial strains including *S.* Enteritidis, *S.* Typhimurium, *C. jejuni*, and *C. perfringens* investigated here.

**Table 2:** Minimum inhibitory concentration (MIC) of peptides that kill 100% bacteria (10^4^–10^5^ CFU/ml). Concentrations are displayed in µM and weight (µg/ml; in the brackets) for each peptide.

AMP	*E. coli* EC317	*Salmonella* Enteritidis LS101	*Salmonella* Typhimurium SL1344	*Campylobacter jejuni* Cj1	*Clostridium perfringens* JP26
CATH-1(6–26)	1.5 (3.75)	1.5 (3.75)	1.5 (3.75)	0.75 (1.875)	1.5 (3.75)
CATH-2(1–15)	7.38 (15)	7.38 (15)	7.38 (15)	0.92 (1.875)	> 118.05 (240)
ABD1	1.66 (7.5)	1.66 (7.5)	1.66 (7.5)	1.66 (7.5)	0.83 (3.75)
ABD2	> 55.50 (240)	> 55.50 (240)	> 55.50 (240)	> 55.50 (240)	> 55.50 (240)
ABD6	> 50.59 (240)	> 50.59 (240)	> 50.59 (240)	> 50.59 (240)	50.59 (240)
ABD9	> 55.97 (240)	> 55.97 (240)	> 55.97 (240)	> 55.97 (240)	> 55.97 (240)

### Cytotoxicity to HD11 cells

**Figure 3:**
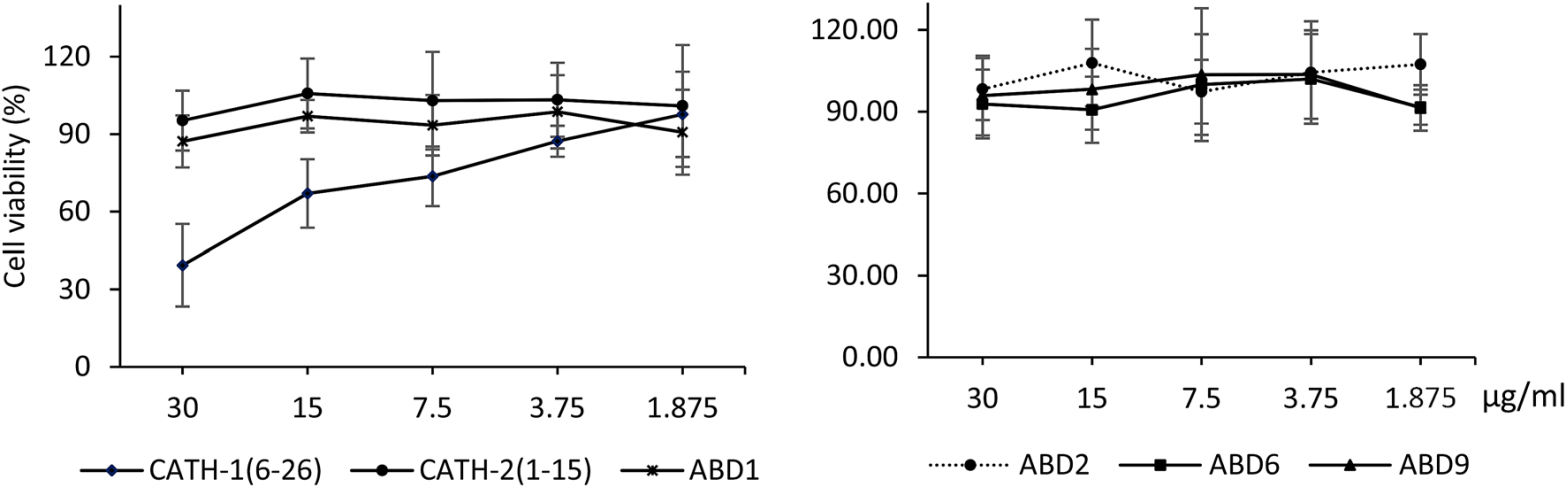
Cytotoxicity of 6 peptides to treated HD11 cells. Cytotoxicity to HD11 cells was determined by WST-1 assay using 1.875–30 µg/ml of each peptide stimulated for 24 h in triplicate. At least three distinct experiments on different days are indicative of the data shown.

To determine the cytotoxic effects of AMPs, HD11 cells were exposed to various concentrations of peptides ranging from 1.875 to 30 µg/ml for 24 h. Within the tested concentrations, five peptides [CATH-2(1–15), ABD1, 2, 6, 9] have not shown any significant effect on the growth and metabolic activity of HD11 cells. However, cells treated with CATH-1(6–26) showed a considerable dose-dependent reduction in metabolic activities and cell viability ranging from 39.33 to 73.66% using a peptide dosage from 30 to 7.5 µg/ml (Fig. 3).

### Chemokines gene expression levels following AMPs stimulation

**Figure 4:**
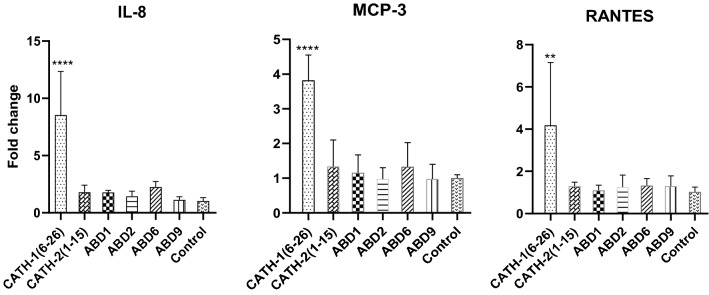
Chemokine induction in HD11 cells post 4 h stimulation of 20 µg/ml synthetic peptides. Data from two separate experiments with three replicates each, means ± SD. Samples from treated groups were compared with the no peptide control using one-way ANOVA with the Dunnett post hoc test. **, **** mean significantly different with *p* < 0.01 and *p* < 0.0001, respectively.

To investigate in vitro transcription of immune response-related genes including IL-8/CXCL-8, MCP-3/CCL7, and RANTES/CCL5, the HD11 cells were exposed to 20 µg/ml of each peptide in triplicate for 4 h. Amongst the peptides studied here, significant up-regulation of these chemokines was observed in cells stimulated by using CATH-1(6–26) (8.55-fold, 3.82-fold, and 4.18-fold for IL-8, MCP-3, and RANTES, respectively). In contrast, there were non-significant differences in gene expression of above-mentioned chemokines in cells stimulated with other peptides and the control groups (Fig. 4).

### NO production and neutralization of LPS-induced NO production in HD11 cells

**Figure 5:**
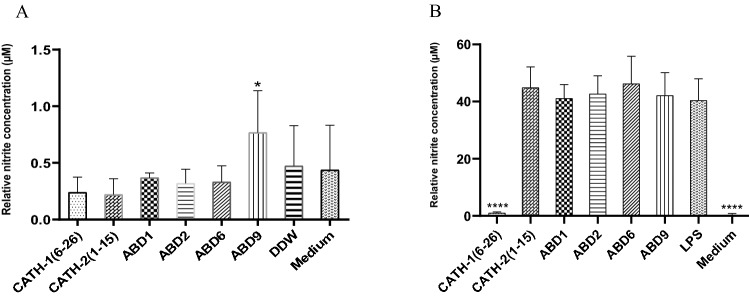
NO production and neutralisation of LPS-induced NO production using the Griess assay. (**A**) NO production by HD11 cells stimulated with 20 µg/ml each peptide after 24 h incubation. * means significantly different between the ABD9 treated and untreated groups (medium group) with *p* < 0.05. (**B**) Neutralization of LPS-induced NO production of HD11 cells by 20 µg/ml of AMPs post 24 h stimulation. **** means significantly different from the LPS-treated group (*p* < 0.0001). One-way ANOVA analysis using the Dunnett *post-hoc* test was used. All data shown are obtained from at least three independent experiments on different days, means ± SD.

NO production was assayed at 24 h post-stimulation of HD11 cells with 20 µg/ml of the respective peptides. Amongst the treatment groups, a significantly higher amount of NO (0.77 µM) was produced in the cells stimulated with ABD9. The cells treated with other peptides showed statistically non-significant differences in NO production (ranging from 0.22 to 0.37 µM) compared to the control groups (non-treated cells; 0.44 µM) (Fig. 5A).

To examine the neutralization of LPS-induced NO production by the peptides, HD11 cells were treated with 20 µg of each peptide pre-mixed (for 15 min) with 100 ng LPS per ml of culture medium. After 24 h incubation with the mixture, NO from culture supernatant was assayed using Griess test as described in the materials and methods section. The results from 3 independent experiments showed a significant inhibition (approximately 97%) of LPS-induced NO production (1 µM) in CATH-1(6–26) treated cells, while other peptides showed non-significant effects on NO production (from 41.13 to 46.24 µM) (Fig. 5B).

### Suppression of LPS-induced IL-1β cytokine gene expression in HD11 cells

**Figure 6:**
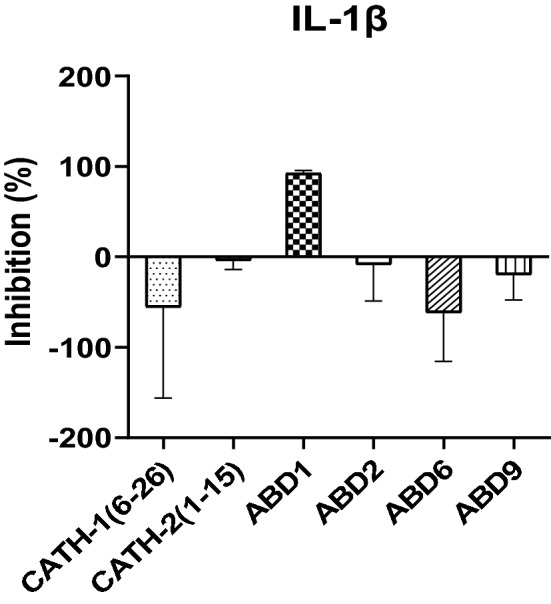
Neutralization of LPS-induced IL-1β production by 20 µg/ml each peptide following 4 h stimulation. Data from two separate experiments with three replicates in each experiment, means ± SD.

Inhibition of LPS-induced IL-1β mRNA expression in HD11 cells was examined by 4 h stimulation of the mixture of 20 µg/ml peptides plus 50 ng LPS. LPS-induced IL-1β gene expression in HD11 cells was blocked by ABD1 (92.4%), while other peptides showed no effect on IL-1β gene expression (Fig. 6).

### In ovo administration of peptides and protection from *E. coli* challenge

**Figure 7:**
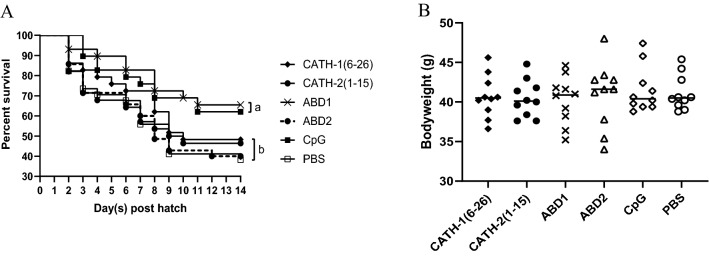
Survival proportions and day-old bodyweight. (**A**) Survival proportions of chickens following *E. coli* challenge (45 CFU/bird via navel route). The statistically significat difference between groups is represented by different letters (a or b). (**B**) Bodyweight of 1-day-old chicks was measured from 10 chicks per group before *E. coli* challenge.

To evaluate potential effects of in ovo administered peptides, following in ovo administration of respective peptides on 18th embryonic day, we measured the bodyweight of 10 hatched chicks from each group. Our results showed non-significant differences in the mean hatching weight of birds amongst all treatment and control groups (Fig. 7B). Furthermore, 1-day post-hatch, the chicks were challenged by using a well-characterized *APEC* strain EC317 via intra-navel route. Prior to *E. coli* challenge, 2 randomly selected newly hatched chicks from each group were confirmed to be free from *E. coli* (samples collected from yolk sac and liver cultured on MacConkey plates). The survival rates of ABD1 (65.52%) and CpG (62.07%) administrated groups exhibited significantly higher protection than the control group (38.24%) at 13 days post-challenge (2 weeks of age). While statically non-significant differences in the survival proportions were found amongst CATH-1(6–26) (48.28%), CATH-2(1–15) (46.43%), ABD2 (40%) groups and the control group (Fig. 7A).

**Figure 8:**
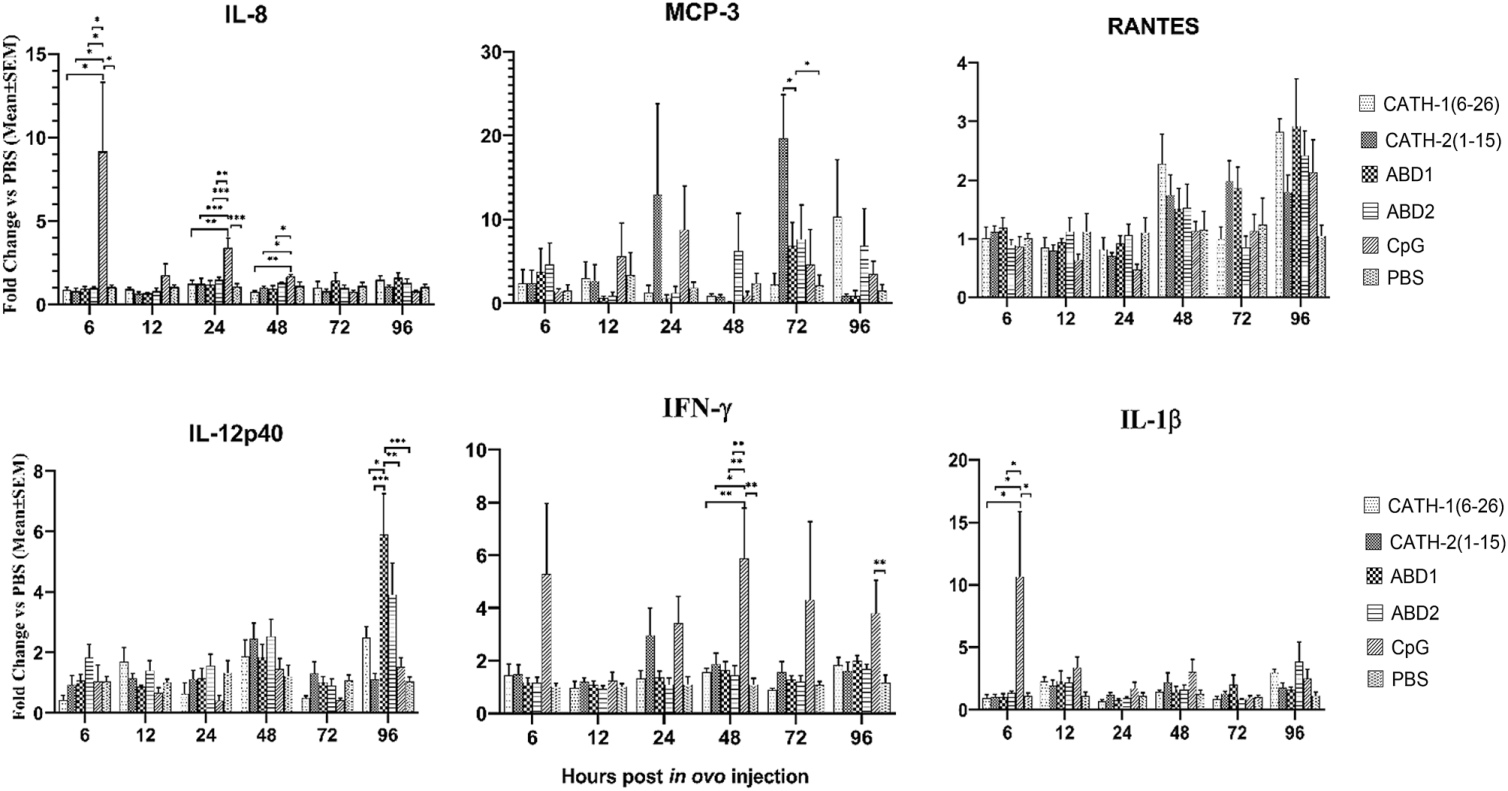
Expression of immune-related genes in spleen tissues post 6, 12, 24, 48, 72, 96 h in ovo treatment with peptides and CpG. Data represent means ± SEM. *, **, *** mean significantly different with *p* < 0.05; *p* < 0.01; *p* < 0.001, respectively.

To elucidate the mechanism of protection offered by the peptides used in these studies, the transcriptional pattern of innate immune response-related cytokines and chemokines genes including IL-12, INF-γ, IL-1β, IL-8, MCP3, and RANTES was monitored in spleen tissues from six embryos at each time point) 6, 12, 24, 48, 72, and 96 h post in ovo treatment. Different gene expression profiles were displayed between CpG and peptide treatment groups. The CpG treated birds showed significant up-regulation of IL-8 at 6, 24, and 48 h, IFN-γ at 48 and 96 h, and IL-1β at 6 h post in ovo treatment. Amongst other treatments, only ABD1 treated chicks showed significant up-regulation of IL-12 gene expression at 96 h, whereas, for all other treatments there were non-significant differences in genes expression between treatments and control groups (Fig. 8).

## Discussions

Innate immune modulators and antimicrobial peptides (AMPs) are drawing considerable attention as potential alternatives to the use of antibiotics. Selection of AMPs as anti-bacterial agents is due to their ability of direct killing of bacteria, immune modulation and non-emergence or emergence of a very low level of bacterial resistance. In the present studies, six avian AMPs were synthesized and characterized for their ability to kill important avian bacterial pathogens, their immune-modulatory effects, and their protective potentials against *E. coli* infection in young chicks. The results from the present study demonstrated that avian AMPs can directly kill bacteria including *E. coli*, *S.* Enteritidis, *S*. Typhymurium, *C. jejuni*, and *C. perfringens* which are associated with human foodborne illnesses and economically important avian infections. Moreover, avian AMPs showed modulation of host innate immunity through differential induction of chemokines, neutralization of LPS-induced IL-1β gene expression, and NO production in HD11 cells. Importantly, some of these synthesized peptides have offered a significant level of protection from the yolk sac infection caused by *APEC*.

AMP’s capacity to kill microbial pathogens is believed to be associated with physicochemical and structural properties of the peptides including cationicity (net charge), hydrophobicity, amphipathicity, length, and α-helicity^25,26^. Net positive charge and hydrophobicity determine the binding and interaction between AMPs and bacterial phospholipid membranes, leading to membrane damage and cell death. The positively charged polar face of AMPs electrostatically interacts with the negatively charged head groups of phospholipids, while the hydrophobicity in nonpolar face allows embedding of AMPs into lipopolysaccharide (LPS) micelles^27,28^. Studies on analogs of certain peptides have shown that the increase in positive charge has an incremental effect on antimicrobial activity with an increase of hemolytic activities^29,30^; whereas, a decrease in hydrophobicity of AMPs has led to reduced hemolytic activities^6^. Increasing α-helicity and hydrophobicity of peptides has shown increased hemolytic and anti-inflammatory activities^6^. Amongst 6 peptides used in these studies, ABD2, possessing the highest level of hydrophobicity (0.616), has displayed the lowest level of antibacterial and cytotoxic activity. CATH-1(6–26) showed higher in vitro antimicrobial activities against all tested bacterial strains though it has a lower positive net charge as compared with CATH-2(1–15) and ABD1. Recently, it has been demonstrated that for efficient binding of AMPs to the bacterial cytoplasmic membrane, a perfect amphipathicity is far more important than hydrophobicity^31^. However, in our studies CATH-2(1–15), despite harboring the highest amphipathicity (hydrophobic moment = 0.585 µM), has lower bactericidal effects in comparison with CATH-1(6–26) and ABD1 with hydrophobic moment = 0.28 µM and 0.181 µM, respectively. From these data, we may assume that anti-microbial potency and targeted selectivity of the peptides may depend on a delicate balance of above-given parameters and may not be correlated with any single physicochemical property of these peptides. Consequently, factors contributing towards differences between the predicted and observed in vitro outcomes may have influenced predicted results following in ovo administration of these peptides.

In in vitro antimicrobial assays, six AMPs indicated varying efficiencies against bacterial strains tested in these studies. The truncated CATH-1(6–26) and ABD1 exhibited highly potent antibacterial activity with MICs ranging from 0.75 to 1.5 µM [CATH-1(6–26)] and 0.83–1.66 µM (ABD1). In agreement with these findings, the previous studies have shown a broad-spectrum antimicrobial activity of CATH-1 against Gram-positive (e.g., *S. aureus*, *L. monocytogenes*) and Gram-negative bacteria (e.g., *E. coli* O157:H7, *S.* Typhimurium DT104, *Klebsiella pneumonia, and Pseudomonas aeruginosa*) with MICs ranging from 0.4 to 5.32 µM^15^. In another report, ABD1 and ABD2 killed 100% of *S. enteriditis*, *C. jejuni*, and *C. albicans* with MICs ranging from 2 to 16 µM^32^. However, in our studies ABD2 was found to be minimally active against avian *E. coli* and *C. jejuni*, and had no effects on other tested strains including *S*. Enteritidis, *S.* Typhymurium, and *C. perfringens*. Amongst various analogs, truncated CATH-2(1–15) has shown to be the most potent inhibitor against *E. coli*, *S. aureus*, *S.* Enteritidis, *B. globigii* with minimal bactericidal concentrations ranging from 2.5 to 7.5 µM^24^. In our studies, CATH-2(1–15) efficiently inhibited the growth of tested Gram-negative bacteria (MIC = 0.92–7.38 µM), however, showed a very low bactericidal activity against, *C. perfringens* (MIC > 118.05 µM). Regarding ABD6 and ABD9, with the exception of a moderate inhibitor activity against *C. perfringens* by ABD6 (MIC = 50.59 µM), there were no effects of these peptides against other tested bacterial strains. In agreement with our findings, in a previous study synthetic and recombinant ABD9 has shown a minimum activity against *S.* Typhymurium, *E. coli*, *S. aureus*, and *Bacillus cereus* (> 128 μM)^19^. Hence, amongst the 6 peptides used here, the CATH-1(6–26), ABD1, and CATH-2(1–15) appear to be the potent peptides for potential use.

A broad-spectrum antimicrobial activity of AMPs makes them an attractive choice as new non-antibiotic therapeutics. However, their application is hindered by their potential toxicity against eukaryotic cells. Owing to non-selective interaction between AMPs and cellular membranes, there is a potential risk of lysis of eukaryotic cells^33^. Thus, an ideal AMP is the one that can offer the maximum antimicrobial activity with a minimum cytotoxicity towards the host. In the present study, CATH-1(6–26) has appeared as the most potent peptide that has shown the highest in vitro antibacterial activity against tested bacteria. However, it also showed the highest toxicity to chicken macrophage cells, HD11, within the concentration of 7.5–30 µg/ml. In contrast, ABD1, the other effective peptide has not caused any negative effects on the survival of HD11 cells within the range of 1.875–30 µg/ml. According to a previous report, the bacterial selectivity of membranes by AMPs is contributed by two important factors: (1) The higher negatively charged fatty acid membrane profile of bacteria compared to eukaryotic host cells, (2) The presence of a large amount of cholesterol, that stabilizes the lipid bilayer of eukaryotic membrane^34^. Hence, the antimicrobial activity of ABD1 with low host cells toxicity suggest a differential membrane selection of microbial cells versus eukaryotic cells. In agreement with our findings about non-toxicity of CATH-2(1–15) treatment of HD11 cells, the CATH-2(1–15) has been previously reported being non-toxic or very mildly toxic towards human peripheral blood mononuclear cells (PBMCs) and chicken erythrocytes^24^. However, in contrast to observed in vitro toxicity of CATH-1(6–26), non-significant differences in weights and hatchability of chicks hatched from CATH-1(6–26) treated and other treatments (with a dosage of 30 µg/embryo) and control group (PBS injected) suggests the absence of toxicity following in ovo administration of CATH-1(6–26).

In addition to the direct antimicrobial activities, many peptides have shown immunomodulatory effects including stimulation of chemotaxis and neutralization of pathogen toxins. Previous studies have shown strong chemotactic activity for neutrophils, but not to monocytes or lymphocytes following CATH-1(6–26) injected into mouse peritoneum^23^. Likewise, in vitro stimulation of human PBMCs with mature CATH-2 or its analog, CATH-2(1–21) for 24 h has resulted in significant induction of monocyte chemotactic protein 1 (MCP-1), while no effect on MCP-1 production was observed with the use of CATH-2(1–15) peptide^24^. Treatment of HD11 cells with full-length CATH-2 has shown dose-dependent induction of chemokines IL-8, MCP3, and RANTES, whereas, there was no induction of IL-1β, a pro-inflammatory cytokine^11^. Our investigations have revealed similar results with significant up-regulation of chemokines IL-8, MCP-3, and RANTES in HD11 cells at 4 h post-stimulation of cells with truncated CATH-1(6–26), whereas, non-significant change in expression of these chemokines was observed in cells stimulated with CATH-2(1–15) and other ABDs peptides. It may be assumed that enhanced expression of these chemokine genes in the CATH-1(6–26)-treated HD11 macrophages might be the cell responses to the potential stressor and toxicity which lead to an approximately 50% cell death post 24 h stimulation. Moreover, it has been said that although three conserved disulfide bridges of β-defensins (Cys1-Cys5, Cys2-Cys4, and Cys3-Cys6) are not likely associated with antimicrobial activity, they are essential for the chemotactic effects^35,36^. Thus, lacking the disulfide connectivity of the linear forms of β-defensins used in our study might have abolished ABDs’ chemotactic activity.

Besides chemotactic cytokines expression, avian AMPs may block NO production and LPS-induced cytokine gene expression. Although antibiotic treatments have saved a number of lives through successfully combating against various infections, yet there are serious concerns about the association of antibiotic therapy with sepsis shock during treatment. Antibiotic mediated killing of bacteria resulting in the accumulation of toxic products such as endotoxin (LPS) may lead to the overwhelming production of inflammatory cytokines, leading to multi-organ dysfunction^37,38^. By contrast, a number of AMPs display a strong affinity to LPS which blocks downstream LPS interaction with the LPS-binding protein (LBP) resulting in suppression of inflammation^12^. Similarly, excessive NO production is known as a major factor in mediating alterations in the vascular system and tissue damage leading to septic shock^39^. Our data suggest that CATH-1(6–26) has the ability to block more than 97% of NO production induced by LPS when this peptide was premixed with LPS prior to cell stimulation. While other peptides used in these studies have not shown this characteristic. As previously stated that CATH-1(6–26) is highly toxic to HD11 cells, we assumed that reduced NO production exhibited by the mixture of CATH-1(6–26) and LPS may be the result of a reduced number of viable cells in the reaction due to cellular toxicity of CATH-1(6–26). However, similar levels of NO production through stimulation of HD11 cells with similar dosages of CATH-1(6–26) and other peptides (with exception of ABD9) without the addition of LPS negated this assumption. Thus, we concluded that CATH-1(6–26) has the ability to neutralize LPS based induction of NO. Furthermore, ABD1 [but not CATH-1(6–26)] has the ability to inhibit LPS-induced IL-1β gene expression following 4 h stimulation (with ABD1 and LPS) of HD11 cells suggests an anti-inflammatory role of ABD1. Despite its capacity to neutralize LPS-induced NO production, CATH-1(6–26) was unable to block IL-1β induction. In conclusion, these data suggest distinctive modes of actions for the LPS neutralization by both of the avian peptides and might not be dependent on the LPS binding affinity alone. Alternatively, we assume that mechanisms for this inhibition may be: (1) inhibition of LPS signaling through binding to cell surface CD14 as observed in human cathelicidin LL-37 and its derivatives^40^, or (2) through inhibition of nuclear translocation of NF-κB subunits p50 and p65, which is pivotal to LPS-induced pro-inflammatory cytokine production as described for LL-37, BMAP-27 and polymyxin B^41,42^.

The strong in vitro bactericidal and immunomodulatory effects of these avian AMPs encouraged us to investigate their in ovo administration for the prevention of early chick mortality due to bacterial infections in young chicks. For in ovo administration, we selected 4 peptides including 3 highly bactericidal in vitro [CATH-1(6–26), CATH-2(1–15), ABD1] and 1 (ABD2) with low in vitro antibacterial activity. Additionally, CpG ODN 2007, a potent innate immune stimulant for the prevention of *E. coli* infection in young chicks, was employed as a positive control. Interestingly, 2 weeks post-challenge, ABD1 showed the highest protective efficacy with a significantly higher survival rate (65.52%) compared with the control group (38%). However, there was a non-significant difference in the protection offered by ABD1 and CpG ODN treated groups. Survival rate (62.%) in the CpG treated group at 14 days post-infection is comparable with previous studies^43,44^. Although high in vitro killing of *E. coli* strain EC317 was exhibited by CATH-1(6–26) and CATH-2(1–15), yet non-significant differences were found in survival rates following in ovo administration of these treatments and PBS treated control group. Similarly, the lowest bacterial scores was displayed in yolk sac and liver samples derived from ABD1 and CpG treated birds (data not shown). Protective effect of ABD1 against MRSA infection has been demonstrated in a mouse model previously^22^. While, this is the first report related to the in ovo protective potential of avian ABD1 showing a reduction of early chick mortality due to *APEC* based YSI. To date, there is only one report about the efficacy of D-analog of chicken cathelicidin-2 (D-CATH-2) tested against respiratory infection of *E. coli*. In referred studies, D-CATH-2 was administrated via the in ovo route and chicks were challenged with a pathogenic *E. coli* strain at 7-days of age^45^. The observation for 7 days following infection showed that in ovo treatment of D-CATH-2 could reduce mortality (30%) and respiratory bacterial load > 90% in comparison with the untreated group^45^. Our results indicated that CATH-1(6–26) is highly bactericidal in vitro, whereas, in ovo administration has shown a significantly lower level of protection compared with ABD1. This discrepancy may be explained by the different immunomodulatory effects, stability, or toxicity of these peptides when injected in ovo. Based on the long interval between in ovo administration of peptides and *E. coli* challenge, the short half-life of AMPs, and in ovo proteolytic degradation of peptides, an extremely low level of peptides is expected to exist in embryonic tissues at the time of *E. coli* challenge. Therefore, the protective effects of ABD1 following in ovo administration are likely by triggering the immunomodulation rather than the direct anti-*E. coli* activity of ABD1.

Innate immune responses mediated through the rapid release of various cytokines and chemokines play crucial roles in inflammation and antibacterial defense. IL-1β, a pro-inflammatory cytokine produced by various cell types including macrophages and monocytes is a key mediator of T-cell proliferation and enhances the production of other cytokines (like IL-6) and chemokines^46^. Similarly, IFN-*γ* is a multifunctional cytokine that has antiviral and antibacterial activities and induces proinflammatory cytokine/chemokine production^47^. IL-12 is produced mainly by dendritic cells and macrophages and is an important immunoregulator of Th1-type immune responses that play roles in IFN-γ production, cell proliferation of chicken splenocytes, and NO production^48^. In the very early stage of infection, chemokines such as IL-8 (CXCL8), MCP-3 (CCL7), and RANTES (CCL5) are produced by numerous cell types. Production of these chemokines plays a significant role in combating pathogens through rapid recruitment of innate immune cells (such as neutrophils or macrophages) at the site of infection. In this study, following in ovo administration of AMPs we monitored the expression of these cytokines and chemokines in spleen tissues of embryos and day-old chicks. Our data shows that in ovo administration of AMPs has a non-significant effect on the expression of above-described cytokines/chemokines genes in spleen cells, except for ABD1, which has shown elevation of IL-12p40 gene expression at 96 h post-injection. While CpG enhanced the expression of various cytokine and chemokine genes including IL-1β, IFN-*γ*, and IL-8 at different time points. Therefore, the mode of stimulating immune responses and protection from bacterial infection by CpG and ABD1 are expected to differ in chick embryos. We may assume that CpG ODN promotes inflammatory responses through the induction of pro-inflammatory cytokines or chemokines, whereas, protection by the ABD1 may be through activation of Th1 type of immune responses associated with IL-12 gene induction.

In conclusion, our studies have shown that six avian AMPs used in this study are able to kill avian pathogens and food-borne illness related pathogens in vitro. Truncated CATH-1(6–26) could stimulate the up-regulation of chemokines such as IL-8, MCP-3, and RANTES in HD11 cells. Besides, CATH-1(6–26) and ABD1 can neutralize LPS-induced NO production and IL-1β expression, respectively to suppress the inflammation. Importantly, ABD1 treatment may offer substantial protection from the yolk sac infection caused by *E. coli.* Moreover, protection from early chick mortality achieved with the use of ABD1 is comparable with the CpG ODN treated group. On the basis of innate immune activation and challenge protection data, we may conclude that two peptides including CATH-1(6–26), and ABD1 may emerge as potential candidates for the replacement of antibiotics in chickens. In general, the data represented here showed that in ovo administration of avian AMPs can be a feasible alternative to antibiotics for the control of bacterial infection in young chicks.

## Methods

### Peptide synthesis and characteristics

All peptides (Table 1) were chemically synthesized in linear forms using the Fmoc solid-phase peptide synthesis (SPPS) method by GenScript, USA. Briefly, to create a peptide chain, SPPS sequentially added amino acids to the 2-Cl Trt resin. On synthesis completion, the Fmoc group’s N-terminal was secured whereas, the side chain protection group was deprotected and the peptide was cleaved off from the resin. Peptide purity was determined by reversed-phase high-performance liquid chromatography (HPLC) and confirmed by mass spectrometry (MS). Lyophilised peptides were stored at – 20 °C and dissolved in LPS-free water (molecular water, Sigma) to obtain working stocks of 1 mg/ml and 3 mg/ml for in vitro and in ovo tests, respectively. To prepare in ovo injectable solution working stocks were diluted in PBS (Sigma) to obtain the peptide concentration of 300 µg/ml.

The 3D structures of the peptides were drawn using the PEP-FOLD3 program (http://bioserv.rpbs.univ-paris-diderot.fr/services/PEP-FOLD3)^49^. The molecular weight and iso-electric point of synthesised peptides were calculated using the peptide property calculator (https://pepcalc.com). The net charge, hydrophobicity, and hydrophobic moment were generated using the online website (http://heliquest.ipmc.cnrs.fr/cgi-bin/ComputParams.py). Weight concentrations of peptides were converted to molar concentrations using an online tool (http://molbiol.edu.ru/eng/scripts/01_04.html).

### Bacterial species and growth conditions

Five bacterial species isolated from birds were used in this study. These five bacterial strains include (1) *E. coli* strain EC317 originally isolated from a case of septicemia in turkey; (2) *S. enterica* serovar Enteritidis (SE) strain LS101; (3) *S.* *enterica* serovar Typhimurium (ST) strain SL1344; (4) *C. jejuni* strain ATCC Cj1 and (5) *C. perfringens* strain JP26 (kindly given by Dr. John Prescott, University of Guelph, Toronto).

The stocks stored at − 80 °C (in culture medium containing 20% glycerol) were revived and used for assays. The *E. coli* and *Salmonella *sp. were cultured on Luria–Bertani (LB) agar and incubated at 37 °C under aerobic conditions; while *C. jejuni* and *C. perfringens* were cultured on Tryptone soy agar plates containing 5% sheep blood (blood agar—BA) and incubated under microaerophilic conditions (10% CO_2_, 5% O_2_ and 85% N_2_) and anaerobic conditions using gaspak (Oxoid), respectively.

### Antibacterial assays

Bacterial killing assays were performed following the methods described by Zhao et al.^50^ and Xiao et al.^21^ with some modification. Briefly, *E. coli* EC317, two colonies picked from an LB plate were sub-cultured in 20 ml Brain Heart Infusion (BHI) broth medium for an additional 3 h at 37 °C to the mid logarithmic phase (OD_600_ = 1.3–1.5, approximately 10^8^ CFU/ml). For *S.* Enteritidis (SE) strain LS101 and *S.* Typhimurium (ST) strain SL1344, two colonies from a Luria–Bertani (LB) plate were sub-cultured in 20 ml LB broth medium for an additional 3 h at 37 °C to the mid logarithmic phase (OD_600_ = 0.7–0.8, approximately 10^8^ CFU/ml). To *C. jejuni* strain Cj1, bacteria were cultured on BA using a cotton swab and incubated at 42 °C in microaerophilic conditions. Bacteria were collected after 20 h incubation and resuspended in Phosphate Buffer Saline (PBS, pH = 7.4, Gibco) to obtain OD600 = 0.35 (approximately 10^8^ CFU/ml). *C. perfringens* strain JP26, a single colony was transferred to 3 ml cooked meat medium (CMM, Difco) and incubated under anaerobic conditions at 37 °C for 24 h. The CMM culture was transferred to fluid thioglycollate broth (FTG, Difco) at a ratio of 1: 30 v/v (JP26: FTG) and incubated under aerobic conditions for 15–16 h at 37 °C (to obtain 10^8^ CFU/ml).

Bacterial suspensions were then diluted in PBS (pH = 7.4, Gibco) to obtain approximately 10^4^–10^5^ CFU/ml. The diluted bacterial suspensions (90 µl) were dispensed into 96-Well Polystyrene Round Bottom Microwell Plates (Thermo Scientific™), followed by the addition of 10 µl of serial twofold dilutions of peptides to achieve concentrations of 240, 120, 60, 30, 15, 7.5, 3.75, 1.875 µg/ml in duplicate. Ten microliters of dilution buffer without peptide were added (in duplicate) as negative controls. The mixtures were incubated for 2 h at 37 °C in suitable conditions for each bacterial type. Surviving bacteria were enumerated after appropriated dilution and plating onto LB plates (for *E. coli* and *Salmonella *sp.), BHI plates (for *C. jejuni* and *C. perfringens*) and incubated overnight.

### HD11 cytotoxicity

Cytotoxicity of six AMPs was evaluated on chicken macrophage cells, HD11, according to Dijk et al. (2009)^24^. Briefly, HD11 cells (passage 2, confluency 80–90%) from T75 flasks were transferred and divided into Nunc 96-well flat-bottom plates to obtain 5 × 10^4^ cells/well/100 µl in RPMI-1640-glutaMAX (Gibco) medium supplemented with 10% fetal bovine serum (FBS) and Gentamicin (Gibco). Cells were grown for 16–18 h at 37 °C in a humidified CO_2_ incubator. The next day, the old medium was replaced with 100 µl of new DMEM/F12 medium (Gibco) (without phenol red, FBS, and Gentamicin) containing 30, 15, 7.5, 3.75, 1.875, 0 µg/ml of each peptide in triplicate. Cells were then incubated at 37 °C in a 5% CO_2_ incubator. After 24 h incubation, old media were replaced with 100 µl of new DMEM/F12 medium (without phenol red, FBS, and Gentamicin) and 10 µl WST-1 (Roche) was added to each well and incubated for 60 min at 37 °C in a 5% CO_2_ incubator. The absorbance was measured at 450 nm using a SPECTRAmax 340 PC Microplate Reader (Molecular Devices, CA, USA). The experiments were repeated at least 3 times on different days.

### Nitric oxide (NO) production assay

Nitrite, a stable metabolite of NO, produced by activated macrophages was measured in cell culture supernatant by the Griess assay (Green et al., 1982)^51^ using (from 0 to 200 μM) sodium nitrite dissolved in fresh DMEM/F12 medium as standards. Briefly, HD11 cells (passage 2, confluency 80–90%) from T75 flasks were transferred into Nunc 48-well plates (1.25 × 10^5^ cells/well/250 µl) in RPMI-1640-glutaMAX medium (supplemented with 10% FBS and Gentamicin). After 16–18 h incubation, the old medium was discarded and cells were treated with a new medium (without FBS and Gentamicin) containing 20 µg/ml of each peptide in the absence or presence of LPS (pre-mixed with 100 ng/ml LPS from *E. coli* O111:B4, Sigma). Cells were grown at 37 °C in a humidified CO_2_ incubator for 24 h. Aliquots of 50 μl supernatant were transferred to the Nunc 96-well flat bottom plates in duplicate for Griess assay following the protocol described by Dijk et al. (2016). Briefly, fifty μl of 1% sulfanilamide (Sigma) (dissolved in 2.5% phosphoric acid) was added to each well and incubated for 5 min at room temperature. Followed by the addition of 50 μl 0.1% *N*-(1-naphthyl) ethylenediamine dihydrochloride (Sigma) and incubated for another 5 min. The optical density at 520 nm was determined using a 96-well microplate reader (SPECTRAmax 340 PC Microplate Reader). NO production was calculated through an equation generated from the standard samples. Each experiment was repeated at least 3 times on different days.

### Gene expression studies

#### HD11 stimulation

For peptide-induced stimulation, the HD11 cells (passage 3, 1.0 × 10^6^ cells/ml) were treated with 20 µg/ml peptides in RPMI-1640 GlutaMax medium for 4 h (at 37 °C, 5% CO2) in triplicate using 12-well plates. For LPS neutralization experiments, a concentration of 50 ng/ml LPS was pre-mixed with or without the addition of 20 µg/ml peptide to the cells, and mixtures were incubated for 4 h. Sterile molecular grade water (Sigma) was added as the negative control in triplicate. The cultured cell suspension was transferred to an Eppendorf tube and centrifuged at 8000×*g* for 5 min to collect the cell pellet. Then, 350 ml of cell lysis buffer (buffer RLT including in RNeasy kit, Qiagen) was added to each well, mixed by pipetting, and transferred to the Eppendorf tube containing cell pellet. Samples were homogenized by vortexing for 1 min and stored at − 80 °C or directly processed for RNA isolation as described below.

#### RNA isolation and cDNA synthesis

The spleens from randomly selected 6 embryos from each in ovo treated and control group were aseptically collected in TRIZOL at 6, 12, 24, 48, 72, and 96 h post in ovo injection. Spleen samples were homogenized and stored at -80 C, until further processing for RNA isolation. RNA from HD11 and spleen cells was isolated using the RNeasy kit (Qiagen) according to the manufacturer's instruction. RNA quality and quantity were determined by the NanoDrop-ND1000 Spectrophotometer (Thermo Scientific™, Canada). To remove injection genomic DNA contamination, isolated RNA samples were treated with DNase I (Thermo Scientific™, Canada) at room temperature for 20 min (10 µl reaction consisting of 1 µg RNA, 1 µl DNase I Reaction Buffer, 1 µl DNase I, and Sigma water). Following incubation, the residual DNase I was inactivated by heating the samples at 65 °C for 10 min with 1 µl EDTA 25 mM. Eleven µl DNase-treated RNA was then used to synthesize cDNA using iScript™ cDNA Synthesis Kit (Bio-Rad Laboratories, Inc.) as per the manufacturer's instruction. The temperature parameters for synthesizing cDNA were as follows: 25 °C for 5 min, 46 °C for 30 min, and terminated by heating at 95 °C for 1 min using a PCR thermal cycler. Quality and successful removal of genomic DNA contamination of the RNA samples were re-evaluated by running on an electrophoresis gel.

#### Quantitative RT-PCR

Quantitative RT-PCR (qRT-PCR) was performed using the respective primers (Table 3) and iQSYBR Green Supermix (Bio-Rad Laboratories, Inc.). The qRT-PCR program was 95 °C for 3 min, followed by 40 cycles of 95 °C for 15 s, 60 °C for 30 s, and 72 °C for 30 s. A 30 min dissociation curve was performed after the last extension step to assess the homogeneity of the PCR product and the presence of primer dimers. qRT-PCR was carried out using a CFX96 System (Bio-Rad Laboratories, Inc.) thermocycler. Target gene expression was normalized to the expression of β-actin as previously described by Livak and Schmittgen^52^.

**Table 3:** Target genes and primer sequences used for quantitative RT-PCR in this study.

Target gene	Primer name	Primer sequence (5′–3′)	References
β-actin	β-actin-F	CAACACAGTGCTGTCTGGTGGTA	^53^
	β-actin-R	ATCGTACTCCTGCTTGCTGATCC	
Ch-IL-8	Ch-IL8-F	CAGCTGCTCTGTCGCAAG	^54^
	Ch-IL8-R	GTGGTGCATCAGAATTGAGCT	
Ch-IL-1β	Ch-IL1β-R	GTTGGAGCGGGCAGTCAG	^54^
	Ch-IL1β-F	GGCATCAAGGGCTACAAGC	
MCP-3	MCP-3F	CTGCTGCTTCTCCTATGTTCAAC	^55^
	MCP-3R	ACACATATCTCCCTCCCTTTCTTG	
RANTES	RANTES-F	CCCTCTCCATCCTCCTGGTT	^11^
	RANTES-R	TATCAGCCCCAAACGGAGAT	
Ch-IFN-γ	Ch-IFN-γ-F	CCAAGAAGATGACTTGCCAGA	^56^
	Ch-IFN-γ-R	ACCTTCTTCACGCCATCAGG	
Ch-IL-12B(p40)	Ch-IL-12B-F	CCGACTGAGATGTTCCTGGA	^56^
	Ch-IL-12B-R	CCTGCACAGAGATCTTGTC	

*Ch-IL* Chicken interleukin, *MCP-3* monocyte chemotactic protein 3, *RANTES* regulated on activation, normal T cell expressed and secreted, *IFN-γ* interferon-gamma.

### In ovo administration of peptides and *E. coli* challenge

#### In ovo administration of peptides

In order to evaluate the protective efficacy of synthesized peptides against YSI by *E. coli*, the peptides including (ABD2) with low in vitro antibacterial activities and CATH-1(6–26), CATH-2(1–15), and ABD1 with high antibacterial activities were selected for in ovo experimental studies. Additionally, CpG ODN 2007 was included as a positive control. A total of 420, 18-day-old live embryos (from Lohmann LSL-lite layers) obtained from breeder operation of the Department of Animal and Poultry Science, University of Saskatchewan, were randomly divided into 6 groups designated as A to F with 70 embryos in each group. Treatment to each group include CATH-1(6–26) (group A), CATH-2(1–15) (group B), ABD1 (group C), ABD2 (group D), and CpG ODN 2007 (group E). Each treatment was administrated in ovo (aimed at the amniotic fluid) at a dose of 30 µg peptide/100 µl PBS/embryo. For the untreated control group (group F), 100 µl of PBS/embryo was injected in ovo*.* To ensure that embryos are free of *E. coli* infection, prior to administration of peptides, yolk sac and liver samples were collected from randomly selected five embryos and cultured on MacConkey plates. After hatch, chicks were shifted to VIDO-InterVac animal care facilities. This work was approved by the University of Saskatchewan’s Animal Research Ethics Board (referred to protocol# 20160079) and adhered to the Canadian Council on Animal Care and Animal Research: Reporting of in vivo Experiments (ARRIVE) guidelines.

#### *E. coli *challenge

Each hatched chick was identified with a neck tag and chicks were placed into an animal isolation room at the Animal Care unit of VIDO, University of Saskatchewan. Prior to the virulent challenge, 10 randomly selected birds from each group were weighed whereas, 2 birds from each group were selected for isolation of *E.coli* from livers and yolk sacs samples. One-day post-hatch, the chicks in each group were challenged with 45 CFU/100 µl of *E. coli* strain EC317 via the intra-navel route as described previously^44^. Following the challenge, all birds were provided with water and commercial chick starter ration (without antibiotics) ad libitum. Birds were examined and clinically scored four times daily at the first 6 days post-challenge (PC) and twice daily thereafter up to 14 days PC. Each bird was assigned a daily clinical score as follows: 0 = normal; 0.5 = slow to move; 1 = ruffled feathers, sitting, reluctant to stand, and mouth breathing; 2 = unable to stand or walk, unable to reach feed or water, wings extended, and difficult breathing; and 3 = found dead. Birds that received a clinical score of 2 were humanely euthanatized (by cervical dislocation)^44^. Euthanatized or dead chicks were necropsied immediately. Yolk sac and liver samples derived from all chicks (dead or euthanized) were swabbed for isolation and identification of *E. coli* by culturing on MacConkey plates.

### Statistical analysis

Data were analyzed using the Prism 8 software (GraphPad Software Inc., San Diego, CA 92108). A comparison of the survival proportions between each treated group to the control group at all time points was performed using the Gehan-Breslow-Wilcoxon test. For NO production and chemokine expressions in HD11 cells, the peptide-treated groups were compared with the no peptide control using one-way ANOVA with the Dunnett post-hoc test. In the test for neutralisation of LPS-induced NO production, data from peptide-LPS treated and non-treated (medium) groups were compared with those from the LPS treated group using one-way ANOVA with the Dunnett test. Statistical differences of gene expressions of various treatments in spleen tissues at each time point were determined using one-way ANOVA (and nonparametric or mixed) with Tukey’s test served as a post-hoc method. Significance was considered at *p* values < 0.05.
